# *Quasi*-In Situ EBSD Investigation of Variant Evolution and Twin Formation in a Hot Isostatic Pressing-Treated Additively-Manufactured Titanium Alloy Under Tensile Loading

**DOI:** 10.3390/ma18133169

**Published:** 2025-07-03

**Authors:** Fengli Zhu, Jiahong Liang, Guojian Cao, Aihan Feng, Hao Wang, Shoujiang Qu, Daolun Chen

**Affiliations:** 1 School of materials Science and Engineering, Nanjing Tech University, Nanjing 211816, China; zhugg@njtech.edu.cn; 2 School of Materials Science and Engineering, Tongji University, Shanghai 201804, China; jiahongliang@tongji.edu.cn (J.L.); aihanfeng@tongji.edu.cn (A.F.); qushoujiang@tongji.edu.cn (S.Q.); 3 Institute of Metal Research, Chinese Academy of Sciences, Shenyang 110016, China; haowang@imr.ac.cn; 4 Department of Mechanical, Industrial and Mechatronics Engineering, Toronto Metropolitan University, Toronto, ON M5B 2K3, Canada

**Keywords:** selective electron beam melting, hot isostatic pressing, α variants, dislocation slip, twinning

## Abstract

The advent of additive manufacturing (AM), also known as 3D printing, has revolutionized the production of titanium alloys, offering significant advantages in fabricating complex geometries with enhanced mechanical properties. This study investigates the variant-specific deformation mechanisms in HIP-treated TA15 (Ti-6.5Al-2Zr-1Mo-1V) titanium alloy, fabricated via selective electron beam melting (SEBM). The alloy exhibits a dual-phase (α+β) microstructure, where six distinct α variants are formed through the β→α phase transformation following the Burgers orientation relationship. Variant selection during AM leads to a non-uniform distribution of these α variants, with α6 (22.3%) dominating due to preferential growth. Analysis of the prismatic slip Schmid factor reveals that α4–α6 variants, with higher Schmid factors (>0.45), primarily undergo prismatic slip, while α1–α3 variants, with lower Schmid factors (<0.3), rely on basal or pyramidal slip and twinning for plastic deformation. In-grain misorientation axis (IGMA) analysis further reveals strain-dependent slip transitions: pyramidal slip is activated in α1–α3 variants at lower strains, while prismatic slip becomes the dominant deformation mechanism in α4–α6 variants at higher strains. Additionally, deformation twins, primarily {10–12}<1–101> extension twins (7.1%), contribute to the plasticity of hard-oriented α variants. These findings significantly enhance the understanding of the orientation-dependent deformation mechanisms in HIPed TA15 alloy and provide a crucial basis for optimizing the performance of additively-manufactured titanium alloys.

## 1. Introduction

Titanium alloys are renowned for their high specific strength and exceptional corrosion resistance, which has led to their widespread use in aerospace, marine, and biomedical applications [1,2,3,4,5]. Among these, TA15 (Ti-6.5Al-2Zr-1Mo-1V) stands out as a near-α titanium alloy. Its superior mechanical properties, thermal stability, corrosion resistance, and high fracture toughness at both room and elevated temperatures have led to its extensive adoption in the aerospace sector [6,7]. The primary hardening mechanism of the TA15 alloy is dislocation strengthening induced by plastic deformation, which contributes significantly to its excellent mechanical performance [8,9]. Traditional manufacturing methods for titanium alloys, such as forging, casting, and rolling, often involve significant material waste, long processing times, and challenges in producing complex structural components [10,11]. Additive manufacturing (AM), also known as 3D printing, presents a compelling alternative, offering significant advantages for fabricating complex titanium alloy components. Specifically, selective electron beam melting (SEBM), which utilizes an electron beam as its energy source, is particularly well-suited for titanium alloy production [12]. SEBM offers several advantages over conventional methods, including streamlined production steps, enhanced material efficiency, improved yield, and greater design flexibility. Its layer-by-layer construction inherently supports the creation of highly geometrically complex components [13]. By carefully controlling the SEBM processing parameters, it is able to produce high-density, high-quality titanium alloys with mechanical properties comparable to, or even surpassing, those of conventionally manufactured materials [13,14,15]. In addition, titanium alloys fabricated via additive manufacturing exhibit excellent biocompatibility, primarily due to the spontaneous formation of a stable TiO_2_ layer, which remains intact and is further enhanced through melting and subsequent hot isostatic pressing, thereby promoting surface densification and oxide regeneration [16,17,18]. Consequently, SEBM has been successfully applied in the production of a variety of titanium alloy products. However, additively manufactured titanium alloys typically contain a high density of pores and microcracks, which necessitate post-processing treatments to enhance their mechanical performance [19,20]. Among various post-processing techniques, hot isostatic pressing (HIP) is particularly effective, as the combined effects of elevated temperature and isostatic pressure not only relieve residual stresses but also promote densification through plastic deformation, creep, and diffusion bonding, thereby efficiently eliminating internal porosity in SEBM-fabricated titanium alloys [21,22].

During the SEBM process, the TA15 alloy is subjected to complex thermal cycles, leading to the formation of unique microstructures. In the β→α phase transformation of TA15, the symmetry of the β phase allows for the formation of 12 distinct α variants. A weak α texture will form if these α variants are generated with equal probability [23]. However, variant selection is a commonly observed phenomenon in titanium alloys produced via additive manufacturing. Previous research has shown that α variant selection is influenced by several factors, including scanning strategy [24], initial β grain orientation [25], grain boundaries [26], residual α phase [27], dislocation defects [28], build size [29], cooling rate [30], holding temperature, and holding time [31]. Stephenson et al. [24] demonstrated that, in linear scanning samples, variant selection is predominantly driven by self-adjustment, while in random scanning samples, it is more significantly influenced by prior β grain boundaries, leading to the formation of macrozones. Furthermore, Zhao et al. [25] showed that the strength of the (110)β texture affects variant selection and, consequently, the final α texture. In the case of selective laser melting (SLM) of Ti-6Al-4V, variant selection during phase transformation follows a self-adjusting process, where three variant clusters form to minimize transformation strain energy [32]. Research on laser solid-forming (LSF) of Ti-6Al-4V has revealed the presence of six distinct α variants in different regions of the deposited sample. Notably, variant selection along the build direction is observed, with the 60°/[11–20] variant dominating the microstructure [33]. In regions with high cooling rates, the length fraction of α/α boundaries with the 63.26°/[–10 5 5 –3] orientation is greater than that of other boundary types. Conversely, in regions with low cooling rates, the 60°/[11–20] α/α boundary predominates. This difference is likely due to the self-adjusting mechanism during the β→α phase transformation [34]. Additionally, it has been reported that in the TA15 alloy, both equiaxed α and lamellar α predominantly deform through basal and prismatic slip modes during the initial stages of deformation. As deformation progresses, multiple slip systems become activated, and pyramidal slip modes appear in grains with high Schmid factors [35].

While extensive research has illuminated the mechanisms governing α phase variant selection and the general slip behavior in additively manufactured titanium alloys [36,37], a significant knowledge gap persists regarding the activation of slip systems in individual α variants during tensile deformation. To bridge this gap, the present study investigates the activation of slip systems in six distinct α variants using *quasi*-in situ room-temperature tensile testing, combined with Electron Backscatter Diffraction (EBSD) and in-grain misorientation axis (IGMA) characterization techniques. The primary objective is to provide a comprehensive understanding of how variant orientation influences the plastic behavior of the α phase, thereby offering valuable theoretical insights for texture optimization and performance-oriented design in the additively-manufactured TA15 alloy.

## 2. Materials and Methods

**Figure 1:**
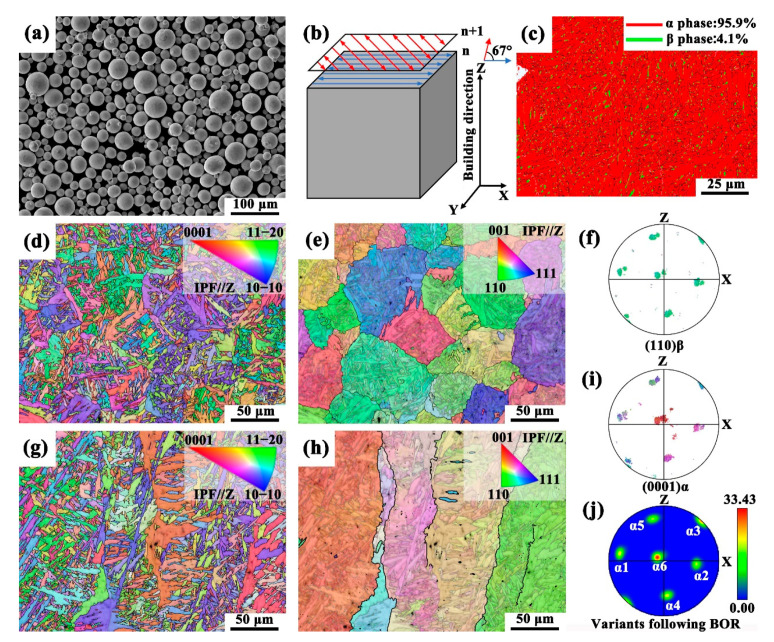
(**a**) Morphology of gas-atomized TA15 powders; (**b**) schematic illustration of the scanning strategy used during SEBM fabrication; (**c**) phase distribution map of the HIPed TA15 alloy; (**d**,**g**) inverse pole figure (IPF) orientation maps of the XOY and XOZ planes of the HIPed TA15 alloy, respectively; (**e**,**h**) reconstructed β grain maps corresponding to (**d**,**g**); (**f**,**i**,**j**) pole figures of the (110)β phase, (0001)α phase, and the six α variants formed according to the Burgers orientation relationship, corresponding to the region shown in (**c**).

The TA15 alloy was fabricated using the SEBM technique, utilizing pre-alloyed powders supplied by AP&C (Saint-Laurent, QC, Canada), as depicted in Figure 1a. Printing was carried out on an Arcam Q20 electron beam additive manufacturing system (Arcam AB, Mölndal, Sweden), with the following key process parameters: a vacuum pressure of 5 × 10^−5^ mbar, an electron beam current of 45 mA, a scan speed factor of 35, a scan spacing of 0.22 mm, and a layer thickness of 0.09 mm. A preheating temperature of 750 °C was maintained, and a scanning strategy involving a 67° rotation between layers was employed (illustrated in Figure 1b). This strategy is known to promote a more isotropic microstructural evolution and improve overall part reliability [38]. The resulting block-shaped samples had dimensions of 150 mm × 20 mm × 100 mm. The samples in the as-printed condition are hereafter referred to as “as-built”. To eliminate metallurgical defects inherent to the additive manufacturing process, a subsequent hot isostatic pressing (HIP) treatment was conducted at 920 °C/120 MPa for 2 h. These HIP-treated samples are subsequently referred to as “HIPed”.

Tensile test specimens, designed in a dog-bone shape with dimensions of 64.4 mm × 12 mm × 2 mm, were prepared from the HIPed blocks using electrical discharge wire cutting. For *quasi*-in situ tensile EBSD testing, the samples were ground using a series of silicon carbide sandpapers (180#, 240#, 400#, 600#, 800#, 1200#, and 2000#), followed by electro-polishing. The electro-polishing was performed with a solution composed of 60% methanol, 34% n-butanol, and 6% perchloric acid (by volume), maintained at –25 °C to –30 °C. A constant current of 0.7–0.8 A was applied with a voltage of 45–60 V for 80–100 s. Tensile testing was performed on an universal testing machine(Instron 5982, Instron, Norwood, MA, USA), with the strain controlled by a contact extensometer at a strain rate of 1 × 10^−3^/s. Each set of tensile tests was repeated three times to ensure accuracy and reproducibility. EBSD characterization was performed using a field-emission scanning electron microscope (Gemini G300, Carl Zeiss, Oberkochen, Germany) equipped with an EBSD detector (Oxford Instruments, High Wycombe, UK). The measurements were carried out at an accelerating voltage of 20 kV, with a 120 μm aperture, a working distance of 12 mm, and a step size of 0.15 μm. The acquired data were subsequently processed and analyzed using Aztec Crystal software (version 2.1). For directional clarity, the deposition direction is designated as the building direction (BD, Z-axis), the initial movement direction on the first powder layer is defined as the X direction, and the direction perpendicular to both of these is the Y direction.

## 3. Results

### 3.1. Initial Microstructure

Figure 1 presents the microstructure of the SEBM-fabricated TA15 alloy after HIP treatment, showing a dual-phase structure comprising α and β phases. The HIP treatment effectively eliminates the inherent metallurgical defects associated with the additive manufacturing process [39]. Additive manufacturing promotes epitaxial growth, resulting in the formation of prior β columnar grains in the HIPed TA15 alloy [40]. As depicted in Figure 1e,h, these prior β columnar grains are reconstructed on the XOY and XOZ planes, respectively, exhibiting size-inhomogeneous prior β columnar grains. During the cooling process inherent to the additive manufacturing of titanium alloys, the α phase precipitates with a specific orientation relationship to the prior β phase: {110}β//{0001}α and <111>β//<11–20>α [41,42]. This crystallographic relationship, known as the Burgers orientation relationship, dictates that the orientation of the α phase is strongly influenced by that of the parent β phase, even if it appears randomly in EBSD maps (Figure 1d,g). Based on this orientation relationship, the α phase can theoretically be categorized into twelve crystallographically distinct variants. However, to simplify the analysis, the α variants were subdivided into six distinct categories based on their crystallographic equivalence and experimental observability in the (0001) pole figure [43,44]. Within a given β (bcc) grain, six different {110}β planes—(1–10), (011), (–101), (110), (0–11), and (101)—can each align with the (0001)α basal plane. This alignment results in the formation of six α (hcp) variants, denoted as α1 to α6, as further illustrated in Figure 1f,i,j and Table 1 [45]. An equal probability of forming these six α variants in each β grain would weaken the α phase texture, thereby reducing the mechanical anisotropy induced by additive manufacturing [46]. Table 1 presents the measured area fractions of the six α variants in the HIPed TA15 alloy, which deviate from the expected area percentage. This deviation is attributed to the variant selective growth during the additive manufacturing process. However, the degree of variant selection in the HIPed TA15 alloy appears to be reduced compared to the as-built condition [47]. This reduction is primarily attributed to the microstructural homogenization induced by the HIP process. The high temperature and isostatic pressure facilitate the release of residual stresses and reduce stored strain energy, thereby diminishing the driving force for the preferential formation of specific α variants. As a result, variant selection becomes less pronounced, and the overall texture intensity is weakened. This observation is consistent with previous reports on HIP-treated additively manufactured titanium alloys [48,49,50]. Given that the HIPed TA15 alloy consists of approximately 95.9% α phase (Figure 1b), its deformation behavior is primarily governed by the α phase, while the residual β phase mainly serves to accommodate strain.

**Figure 2:**
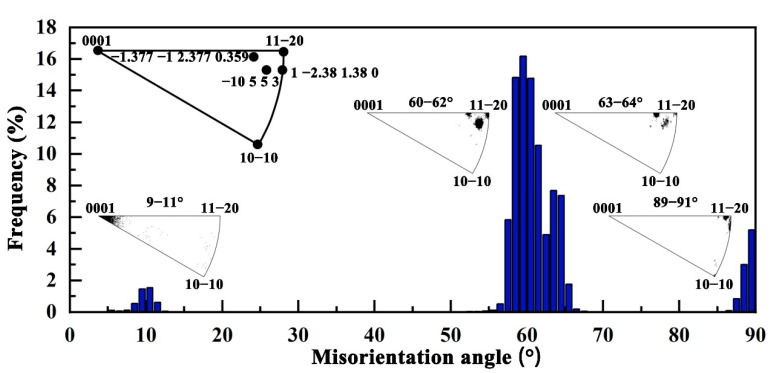
Misorientation angle distribution of the HIPed TA15 alloy measured on the XOZ plane.

An alternative approach to identifying variant selection involves the analysis of α/α boundaries. Figure 2 presents the distribution of misorientation angles and axes between α grains in the HIPed TA15 alloy, revealing four pronounced peaks at approximately 10°, 60°, 63°, and 90°, which are attributed to misorientations between different α/α variant boundaries. According to the Burgers orientation relationship, any two different α variants can form five types of α/α boundaries, expressed as axis–angle pairs: [0001]/10.53°, [11–20]/60°, [1.377 −1 2.377 0.359]/60.83°, [−10 5 5 3]/63.26°, and [1 2.38 −1.38 0]/90° [47,51]. The theoretical fractions of these boundary types are 9.1%, 18.2%, 36.4%, 18.2%, and 18.2%, respectively [23]. In contrast, the corresponding measured fractions are 3.6%, 45.8%, 15.4%, 19.9%, and 5.2%. The significant deviations between the theoretical and experimental distributions are notable. Boundaries corresponding to [11–20]/60°, [−1.377 −1 2.377 0.359]/60.83°, and [−10 5 5 3]/63.26° are observed to be notably more frequent, while [1 2.38 −1.38 0]/90° is less prevalent. These deviations further confirm the presence of variant selection in the HIPed TA15 alloy. The selective growth of variants highlights the necessity of investigating the deformation behavior of individual variants. Ultimately, the basket-weave microstructure, formed by the interlacing of the six α variants, constitutes the fundamental origin of the mechanical properties observed in the HIPed TA15 alloy.

### 3.2. Quasi-In Situ EBSD

**Figure 3:**
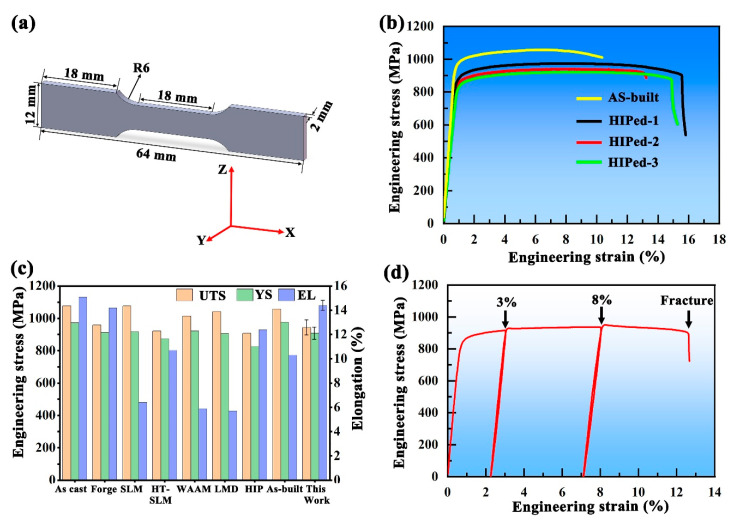
(**a**) Geometry and dimensions of the *quasi*-in situ tensile EBSD sample; (**b**) engineering stress–strain curve of the as-built and HIPed TA15 alloy (three repeated tests); (**c**) comparison of the tensile properties of the TA15 alloy fabricated by different techniques and heat treatment [52,53,54,55,56,57,58]; (**d**) engineering stress–strain curve of the HIPed TA15 alloy during the *quasi*-in situ tensile test. HT-SLM: SLM-fabricated TA15 alloy subjected to post-process heat treatment; WAAM: wire arc additive manufacturing; LMD: laser metal deposition.

As shown in Figure 3b, the HIPed TA15 alloy exhibits a yield strength (YS) of 909 ± 26 MPa, an ultimate tensile strength (UTS) of 943 ± 27 MPa, and a fracture elongation (EL) of 14.4 ± 1.4%. In contrast, the as-built alloy shows a higher YS of 975 MPa and UTS of 1057 MPa, but a lower EL of 10.3%. These results demonstrate that the HIP process enables a strength–ductility trade-off, with a moderate reduction in strength accompanied by a notable improvement in ductility. In a comparison of the mechanical properties of the TA15 alloy with different techniques, as presented in Figure 3c [52,53,54,55,56,57,58], the HIPed TA15 samples exhibit a reduced strength but significantly enhanced ductility, resulting in an improved strength–ductility synergy. This enhancement primarily results from two counteracting effects: the detrimental coarsening of the α variants, accompanied by a reduction in dislocation density, which tends to reduce strength, and the intentional annihilation of defects during the HIP process, which significantly improves plasticity [48,49]. The variation in fracture elongation among the HIPed samples can be attributed to subtle differences in local Schmid factor distributions and α variant configurations, which affect the activation of slip systems and deformation coordination. These microstructural factors influence strain localization and crack propagation behavior, thereby contributing to the observed minor differences in ductility. Compared with annealed TA15 samples fabricated via selective laser melting, the HIPed TA15 alloy exhibits a comparable strength level but demonstrates a markedly improved fracture elongation [58]. Figure 3d presents the engineering stress–strain curve of the HIPed TA15 alloys during *quasi*-in situ tensile testing. The black arrows on the curve indicate the specific strain value at which EBSD data were acquired.

**Figure 4:**
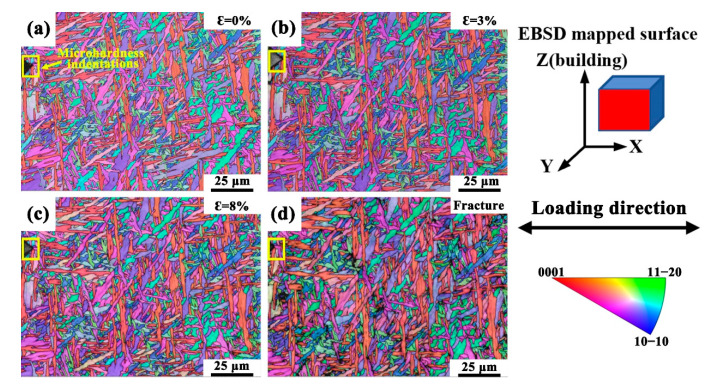
(**a**–**d**) EBSD IPF orientation maps of the HIPed TA15 alloy obtained by EBSD on the XOZ plane during *quasi*-in situ tensile deformation at strain levels of 0%, 3%, 8%, and at fracture, respectively. The yellow frame outlines the region where microhardness indentations were applied for marking purposes.

Figure 4 shows the EBSD inverse pole figure (IPF) orientation maps captured during the *quasi*-in situ tensile deformation of the HIPed TA15 alloy, illustrating the microstructural evolution throughout deformation at various strain levels: 0%, 3%, 8%, and at fracture. During tensile loading, the overall grain morphology remains largely unchanged, although slight grain rotation is observed. This indicates the occurrence of dislocation slip and grain-to-grain cooperative deformation, which collectively induce grain rotation in the HIPed TA15 alloy. Although the influence of a free surface may affect the local stress state during electron microscopy-based tensile experiments, previous studies [59,60,61] have demonstrated that in situ EBSD observations can still reliably capture the evolution of the microstructure in titanium alloys. Furthermore, the continuous and coherent changes in crystallographic orientation with increasing strain further affirm the reliability of the deformation behavior captured.

### 3.3. Slip Mechanisms of α Variants

**Figure 5:**
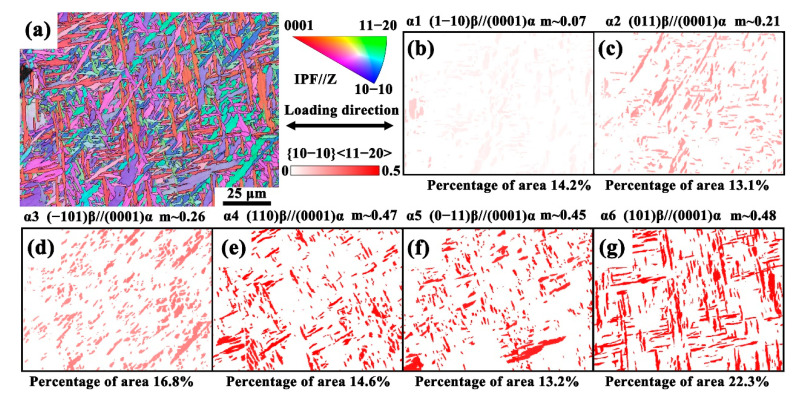
(**a**) EBSD IPF orientation map of the HIPed TA15 alloy at 0% strain on the XOZ plane; (**b**–**g**) corresponding Schmid factor maps of the α1–α6 variants under prismatic slip, determined according to the Burgers orientation relationship.

Considering that the critical resolved shear stress (CRSS) for the prismatic slip system in hexagonal close-packed (HCP) crystal structures of α titanium alloys is the lowest (Table 2), making it the most common slip system for these alloys, the prismatic Schmid factors for different variants were extracted to analyze their individual variant slip behavior [62,63]. Figure 5 presents the EBSD IPF orientation map of HIPed TA15 at 0% strain, along with the prismatic Schmid factors for the six α variants (α1–α6). The prismatic slip Schmid factors for the six α variants {0001}<11–20> are distinctly different. This difference indicates that, when subjected to the same load, different variants possess varying slip resistances, thereby influencing the overall deformation behavior and mechanical properties of the HIPed TA15 alloy [64]. More specifically, the prismatic slip Schmid factors for α1–α3 are relatively low (below 0.3), and their inherently higher slip resistance requires the activation of other slip systems or twin formation during deformation. In contrast, the prismatic slip Schmid factors for α4–α6 are higher (greater than 0.45), allowing these variants to primarily undergo plastic deformation through prismatic slip.

**Figure 6:**
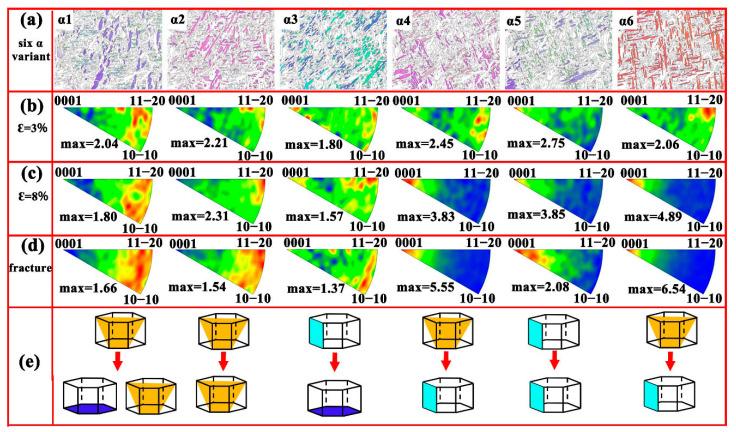
IGMA analysis of the six α variants in the HIPed TA15 alloy on the XOZ plane: (**a**) EBSD IPF orientation maps of the six variants; (**b**–**d**) corresponding IGMA maps at strain levels of 3%, 8%, and at fracture, respectively; (**e**) schematic illustration of various slip planes in the HCP unit cell.

The in-grain misorientation axis (IGMA) method, based on EBSD data, analyzes the crystallographic orientation differences between different regions within the same grain, revealing the microstructural evolution and local orientation changes within grains [65]. This method provides valuable insight into the internal stress development, twinning behavior, and slip mechanisms in HCP alloys [66,67,68]. Figure 6 shows the IGMA distribution results for different α variants in the HIPed TA15 alloy at various strain stages. At 3% strain (Figure 6b), the Taylor axis distributions of α1, α2, and α4 are predominantly aligned at <5 8 –13 3>, indicating that the primary activated slip system is the pyramidal Ⅱ <*c+a*> slip [69]. In contrast, the Taylor axis distribution of α3 is more scattered, making it difficult to identify the dominant slip system. Meanwhile, the Taylor axis of α5 and α6 variants is strongly oriented along the <0001> and <11–20> axes, respectively, indicating that the activated slip system for α5 is the prismatic <*a*> slip, while α6 engages pyramidal Ⅰ <*a*> slip. At 8% strain, the Taylor axis of α1 shifts towards the <10–10> and <11–20> axes, suggesting the activation of both basal *<a>* slip and pyramidal Ⅰ <*a*> slip. Similarly, the Taylor axis of α2 and α3 variants becomes concentrated along <11–20>, signifying that the pyramidal Ⅰ <*a*> slip is activated and becomes the dominant slip system for α2. For α4, α5, and α6 variants, the Taylor axis shifts from <11–20> to <0001>, reflecting a transition from pyramidal Ⅰ <*a*> slip to prismatic <*a*> slip as the prevailing slip mode. Upon fracture, the Taylor axis distributions of all the six α variants closely resemble those observed at 8% strain, reinforcing the observed deformation trends. In summary, during deformation, the α1, α2, and α4 variants primarily activate pyramidal Ⅱ <*c+a*> slip, while the α5 and α6 variants activate prismatic <*a*> slip and pyramidal Ⅰ <*a*> slip. As the strain increases, α1 also activates basal <*a*> slip and pyramidal Ⅰ <*a*> slip, while pyramidal Ⅰ <*a*> slip becomes dominant in α2 and α3. Ultimately, by the end of deformation, the α4, α5, and α6 variants transition to prismatic <*a*> slip as the main slip system.

**Figure 7:**
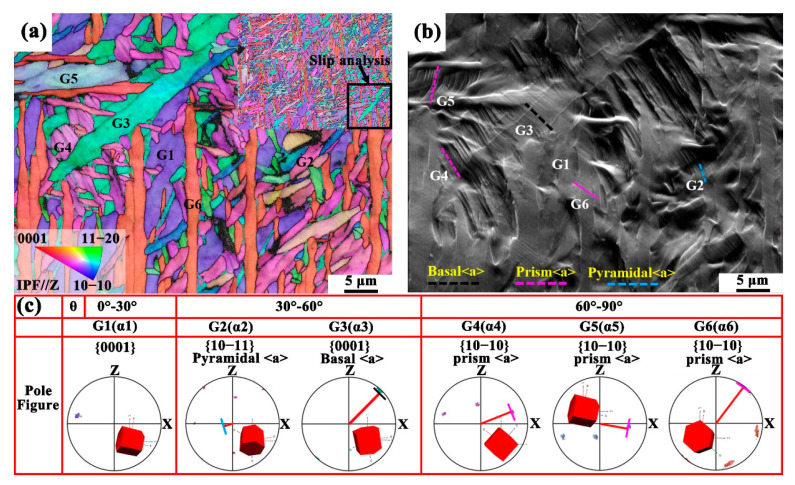
(**a**,**b**) EBSD IPF orientation map and FSD image of the HIPed TA15 alloy at the fracture stage, measured on the XOZ plane. The inset in (**a**) shows the specific region where the IPF map was acquired. (**c**) Schematic diagram illustrating the trace analysis method.

The slip behavior of different α variants in the HIPed TA15 alloy after fracture was analyzed using the slip trace method and subsequently verified with the IGMA results. The six α variants were categorized and integrated according to the misorientation angle (*θ*) between the c-axis of the grain and the loading direction (LD). As depicted in Figure 7(a), grains G1 to G6 corresponded to α1 to α6, respectively, and were classified into three *θ* intervals: 0–30° (G1), 30–60° (G2–G3), and 60–90° (G4–G6). Figure 7b presents the FSD (forward scatter detector) map, where slip systems for six grains are identified using slip traces. It was observed that the activation of specific slip systems is directly related to the angle (*θ*) between the c-axis of the variants and the loading direction. For the α1 variant (G1) with *θ* in the 0–30° range, no slip traces were observed on the grain surface, indicating that this orientation represents a hard orientation, where slip is difficult to activate [70]. At *θ* = 30–60°, the α2 variant (G2) activates pyramidal slip, while the α3 variant (G3) exhibits characteristics of basal slip. Notably, for grains G4–G6 (corresponding to α4−α6) with *θ* = 60–90°, dense slip traces identified as prismatic slip were observed on the surface. This observation is consistent with the results from the IGMA analysis. The room-temperature tensile deformation mechanism of the HIPed TA15 alloy demonstrates significant orientation dependence. For the α4–α6 variants, deformation predominantly occurs via prismatic slip. This is due to the limited number of available slip systems for basal slip and the higher critical resolved shear stress (CRSS) required for pyramidal slip. In these variants, basal slip and pyramidal slip act as coordinative deformation mechanisms. However, for the α1–α3 variants, plastic deformation is primarily accommodated by other slip systems or deformation mechanisms because of the lower Schmid factor for prismatic slip. Specifically, pyramidal slip and basal slip are activated for the α2 and α3 variants, respectively. The crystallographic orientation heterogeneity among the α variants within individual β grains triggers the coordinated activation of multiple slip systems, thereby promoting strain accommodation behavior that effectively mitigates stress concentration through enhanced deformation compatibility.

### 3.4. Twin Mechanisms

**Figure 8:**
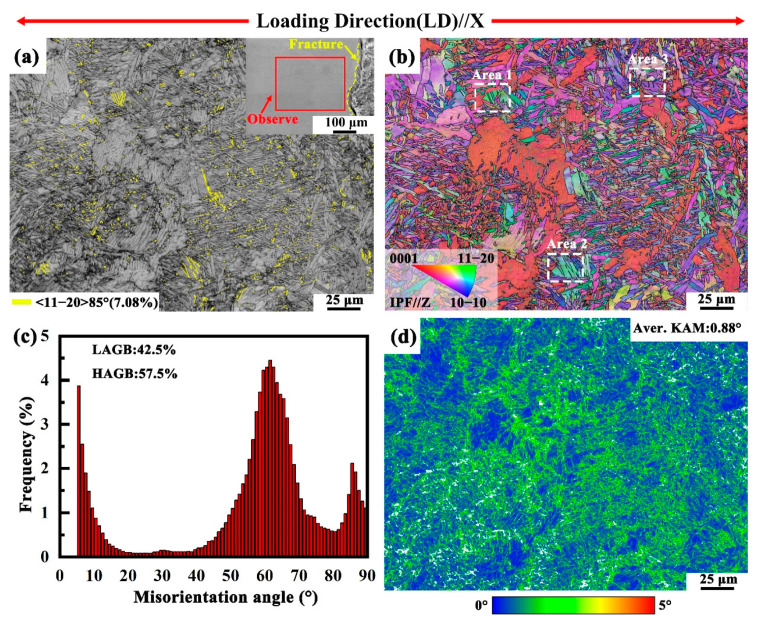
(**a**,**b**,**d**) BC map, EBSD IPF orientation map, and KAM map of the fracture region of the HIPed TA15 alloy after *quasi*-in situ tensile testing, measured on the XOZ plane; (**c**) misorientation angle distribution.

The α1 variant exhibits a low prismatic slip Schmid factor and shows no observable slip traces, suggesting that plastic deformation in this variant is difficult to achieve solely through dislocation slip. In addition to dislocation slip, twinning also plays a significant role in the plastic deformation of titanium alloys [71,72,73,74]. Deformation twins in α titanium alloys can be classified into extension and contraction twins. Studies indicate that when the c-axis of α titanium is subjected to tensile stress, the formation of {10–12}<1–101> and {11–21}<1–126> extension twins is favored. Conversely, when the c-axis is subjected to compressive stress, {11–22}<1–123> and {-1–10–1}<1–012> contraction twins are more likely to form [75,76]. Figure 8 presents the EBSD map from the fracture region of the HIPed TA15 alloy, with the observation area indicated in Figure 8a. Figure 8a also displays the boundary map (BC) of the fracture region, where yellow boundaries represent {10–12}<1–101> 85° extension twins [77]. A statistical analysis of the deformation twin boundaries reveals that the content of {10–12}<1–101> 85° deformation twin boundaries is approximately 7.1%, suggesting that dislocation slip predominates in the plastic deformation process of the HIPed TA15 alloy, with deformation twins playing a coordinating role.

Figure 8b shows the EBSD IPF orientation map of the fracture location, revealing consistent local orientation macrozones and interwoven basket-like structures [78,79]. It is evident from Figure 8a that the {10–12}<1–101> 85° extension twins primarily reside within the basket-like structure, while the α colonies exhibit a lower twin density. Figure 8c presents the corresponding misorientation angle distribution. A significant increase in low-angle grain boundaries is observed, while the 60.8° and 63.22° grain boundaries are notably reduced. This phenomenon is attributed to the adjustments following the Burgers relationship between the α phases and prior-β phase during plastic deformation of the HIPed TA15 alloy, leading to the annihilation of some 63.22°/[10 5 5 3] interfaces [80]. The formation of extension twins results in a slight increase in 85° twin boundaries. Figure 8d shows the corresponding Kernel average misorientation (KAM) map, where higher KAM values are observed at the α phase grain boundaries, and their distribution is relatively uniform. The low overall KAM value (0.88°) indicates limited lattice misorientation and enhanced strain homogeneity, which is closely associated with the coordinated deformation behavior of α variants.

**Figure 9:**
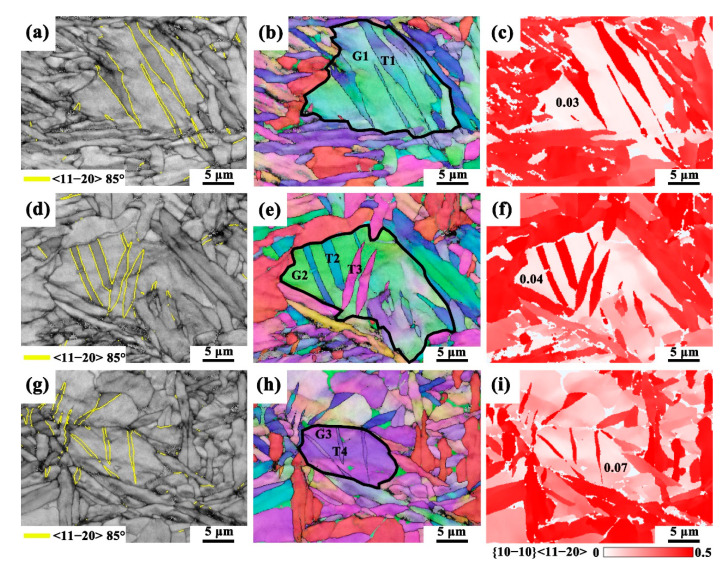
(**a**–**c**) BC map, EBSD IPF orientation map, and Schmid factor map of Area 1 in Figure 8b; (**d**–**f**) BC map, IPF map, and Schmid factor map of Area 2 in Figure 8b; (**g**–**i**) BC map, IPF map, and Schmid factor map of Area 3 in Figure 8b of the HIPed TA15 alloy, all measured on the XOZ plane.

**Figure 10:**
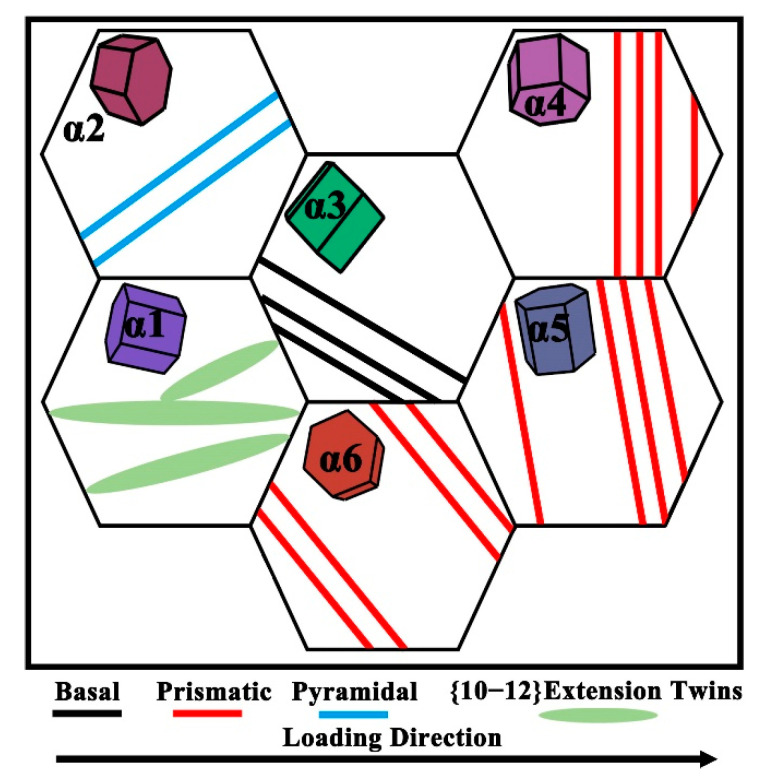
Schematic illustration of deformation mechanisms in α variants.

To better understand the deformation mechanism of extension twinning, three representative twinning regions (Area 1, Area 2, and Area 3) from Figure 8b were selected for detailed analysis, as presented in Figure 9. Figure 9 displays the BC maps, IPF orientation maps (along the Z-axis), and the corresponding Schmid factor maps in these three areas. From the BC map in Figure 9a, various sizes of {10–12}<1–101> 85° extension twins are observed. Figure 9c shows the prismatic slip Schmid factor, which is notably low, with a value of only 0.03, indicating that the parent grain possesses a hard orientation, making dislocation slip difficult. Consequently, to accommodate the applied deformation and relieve local strain, twinning occurs within the grain [81]. Similar observations were made in Area 2 and Area 3, consistently confirming that, in the plastic deformation of the HIPed TA15 alloy, twinning primarily arises due to a low prismatic slip Schmid factor, which makes dislocation slip more difficult [82]. As a result, twinning emerges as the dominant deformation mechanism. In α1 variants exhibiting low Schmid factors, twinning-mediated stress relaxation effectively alleviates localized stress concentrations, thereby enhancing strain accommodation and promoting deformation homogeneity. As schematically illustrated in Figure 10, the deformation of the HIPed TA15 alloy can be categorized into two types: First, grains with low prismatic slip Schmid factors (α1–α3 variants) deform plastically through twinning or activation of cone and basal slip. Second, grains with high prismatic slip Schmid factors (α4–α6 variants) deform plastically through prismatic slip. This coordinated twin–slip deformation mechanism contributes to the simultaneous enhancement of both ductility and strength in the TA15 titanium alloy.

## 4. Conclusions

The microstructure and deformation behavior of α variants in the TA15 alloy, fabricated using selective electron beam melting (SEBM) and post-treated by hot isostatic pressing (HIP), were investigated through *quasi*-in situ EBSD. This comprehensive analysis led to the following main conclusions:

(1) In the variant selection of the SEBM-fabricated TA15 alloy followed by HIP treatment, all six α variants were observed. Notably, the area percentage of the α6 variant was significantly higher than the theoretical value. Additionally, the length fraction of Type II ([11–20]/60°) α/α boundaries was higher than that of other boundary types.
(2) The activation of slip systems within the β grain varies among different α variants. Through IGMA and slip trace analyses, it was found that the α2 variant predominantly activates pyramidal slip, the α3 variant primarily activates basal slip, and the α4–α6 variants largely activate prismatic slip. Conversely, the α1 variant demonstrates difficulty in slip activation.
(3) The activation of {10–12}<10–11> extension twins is strongly influenced by grain orientation. Specifically, when the c-axis of α1 variants aligns parallel to the tensile loading direction, the resolved tensile stress along the c-axis is maximized. This orientation minimizes the critical resolved shear stress required for twinning, thereby facilitating the formation of {10–12}<10–11> extension twins.

## Figures and Tables

**Table 1:** Classification and area fractions of the six α variants in the HIPed TA15 alloy.

Variant		Plane Parallel	Direction Parallel	Percentage of Area	
α1	V1	(1–10)β//(0001)α	[111]β//[11–20]α	9.3%	14.2%
	V2	(1–10)β//(0001)α	[11–1]β//[11–20]α	4.9%	
α2	V3	(011)β//(0001)α	[11–1]β//[11–20]α	8.0%	13.1%
	V4	(011)β//(0001)α	[1–11]β//[11–20]α	5.1%	
α3	V5	(10–1)β//(0001)α	[1–11]β//[[11–20]α	9.4%	16.8%
	V6	(10–1)β//(0001)α	[111]β//[11–20]α	7.4%	
α4	V7	(110)β//(0001)α	[1–11]β//[11–20]α	10.0	14.6%
	V8	(110)β//(0001)α	[–111]β//[[11–20]α	4.6%	
α5	V9	(01–1)β//(0001)α	[–111]β//[11–20]α	5.4%	13.2%
	V10	(01–1)β//(0001)α	[111]β//[11–20]α	7.8%	
α6	V11	(101)β//(0001)α	[11–1]β//[11–20]α	14.4%	22.3%
	V12	(101)β//(0001)α	[–111]β//[11–20]α	7.9%	

**Table 2:** Schmid factors for the slip systems of α1–α6 variants, including prismatic <*a*>, basal <*a*>, pyramidal I <*a*>, and pyramidal II <*c+a*> types, along with the critical resolved shear stresses (CRSSs) [62,63].

Slip Mode	Slip Plane	α1	α2	α3	α4	α5	α6	CRSS
Prismatic <*a*>	{10–10}<11–20>	0.07	0.21	0.26	0.47	0.45	0.48	96 MPa
Basal <*a*>	{0001}<11–20>	0.34	0.46	0.48	0.07	0.23	0.15	127 MPa
Pyramidal Ⅰ <*a*>	{10–11}<11–20>	0.19	0.36	0.39	0.43	0.46	0.47	140 MPa
Pyramidal Ⅱ <*c+a*>	{10–11}<11–2–3>	0.49	0.42	0.37	0.45	0.48	0.48	240 MPa

## Data Availability

The original contributions presented in this study are included in the article material. Further inquiries can be directed to the corresponding author.

## Abbreviations

The following abbreviations are used in this manuscript:

AM	Additive manufacturing
HIP	Hot isostatic pressing
SEBM	Selective electron beam melting
IGMA	In-grain misorientation axis
EBSD	Electron Backscatter Diffraction
CRSS	Critical resolved shear stress
IPF	Inverse pole figure
BC	Band contrast
FSD	Forward scattered detector
KAM	Kernel average misorientation
